# 925. Infectious Complications Following Chimeric Antigen Receptor (CAR) T-cell Therapy

**DOI:** 10.1093/ofid/ofab466.1120

**Published:** 2021-12-04

**Authors:** Caitlin Trottier, Christian Larsen, Poorva Bindal, Laura E Dodge, Pavania Elavalakanar, Elisabeth Knudsen, Sirwoo Kim, Emma Logan, Arielle R Urman, Matthew Frigault, Jay A Fishman, Jay A Fishman, Jon Arnason, Carolyn D Alonso

**Affiliations:** 1 Beth Israel Deaconess Medical Center, Boston, Massachusetts; 2 Massachusetts General Hospital, Boston, Massachusetts; 3 BIDMC, Boston, Massachusetts; 4 Massachusetts General Hospital - Harvard Medical School, Boston, MA

## Abstract

**Background:**

Chimeric antigen receptor (CAR-T) T-cell therapy is a novel immunotherapy for cancer treatment in which patients are treated with targeted, genetically-modified T-cells. Common side effects include cytokine release syndrome, neurotoxicity, hypogammaglobulinemia, and increased susceptibility to infections. Long-term infectious outcomes are poorly characterized.

**Methods:**

We retrospectively examined patients who received CAR-T therapy at BIDMC & MGH from July 2016 to March 2020 and evaluated bacterial, fungal, viral, and parasitic infections at 3 months intervals to 1 year following cell infusion. The incidence, timing, and outcomes of the infectious complications were evaluated.

**Results:**

In total, there were 47 patients; averaging 61.4 years of age (±12 years). Primary indications for CAR-T therapy included diffuse large b-cell lymphoma (65%) and multiple myeloma (25%), chronic lymphocytic leukemia (2%) and mantle cell lymphoma (2%). Patients had received an average 4 ± 2.9 lines of chemotherapy prior to CAR-T infusion; 19 subjects (40%) had a history of prior autologous stem cell transplant. All patients received acyclovir for antiviral prophylaxis and most received either trimethoprim-sulfamethoxazole (24/47; 51%) or atovaquone (16/47; 34%) for pneumocystis prophylaxis. In the first year, 35/47 (74.5%) of subjects experienced at least one infection with an infection rate of 84.4/10,000 person days. Median time to first infection was 59 days (range 1-338 patient days). 31/47 (66.0%) subjects had at least one bacterial infection, with pulmonary (42/113; 37.2%) sources being the most common site of infection. 13/47 (27.7%) of patients had a viral infection (predominantly respiratory viral infections) and 6/47 (12.8%) had a proven or probable fungal infection. Death attributed to infection was noted in 2 subjects (4.3%), both related to COVID-19. Baseline IgG levels were significantly lower in the group with infections (p=0.028), while white blood cell count and absolute neutrophil counts were comparable.

Table 1. Baseline Demographic, Clinical Characteristics, and Outcomes of 47 Recipients of CAR-T Cell Therapy by Infection Status

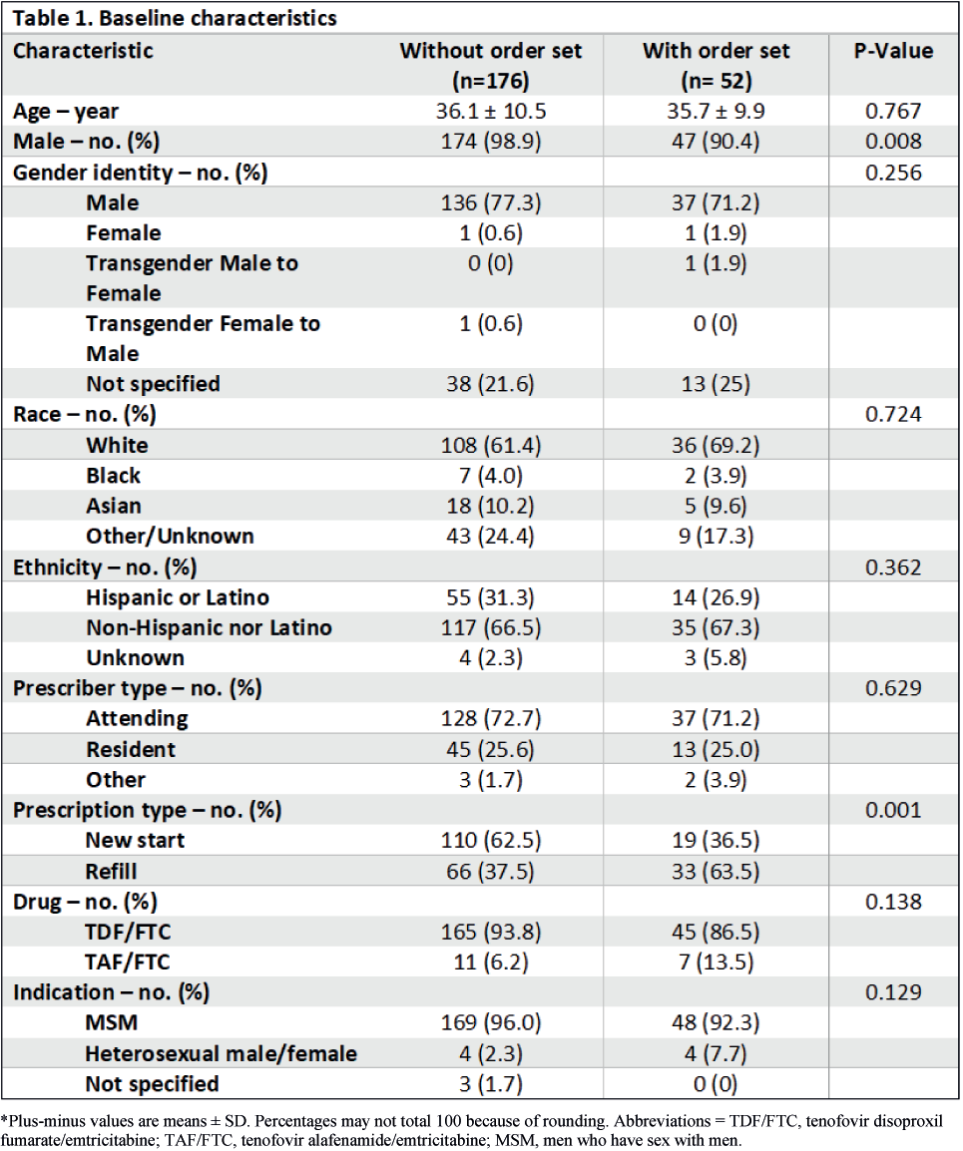

**Notes:**

BMI: body mass index; DLBCL: diffuse large B-cell lymphoma; CLL: chronic lymphocytic leukemia; Flu/Cy: Fludarabine/cyclophosphamide; IVIG: intravenous immunoglobulin; WBC: white blood cell count; ANC: absolute neutrophil count; ALC: absolute lymphocyte count.

Table 2. Characteristics of the 113 Infections in the 35 Subjects Who Developed Infections

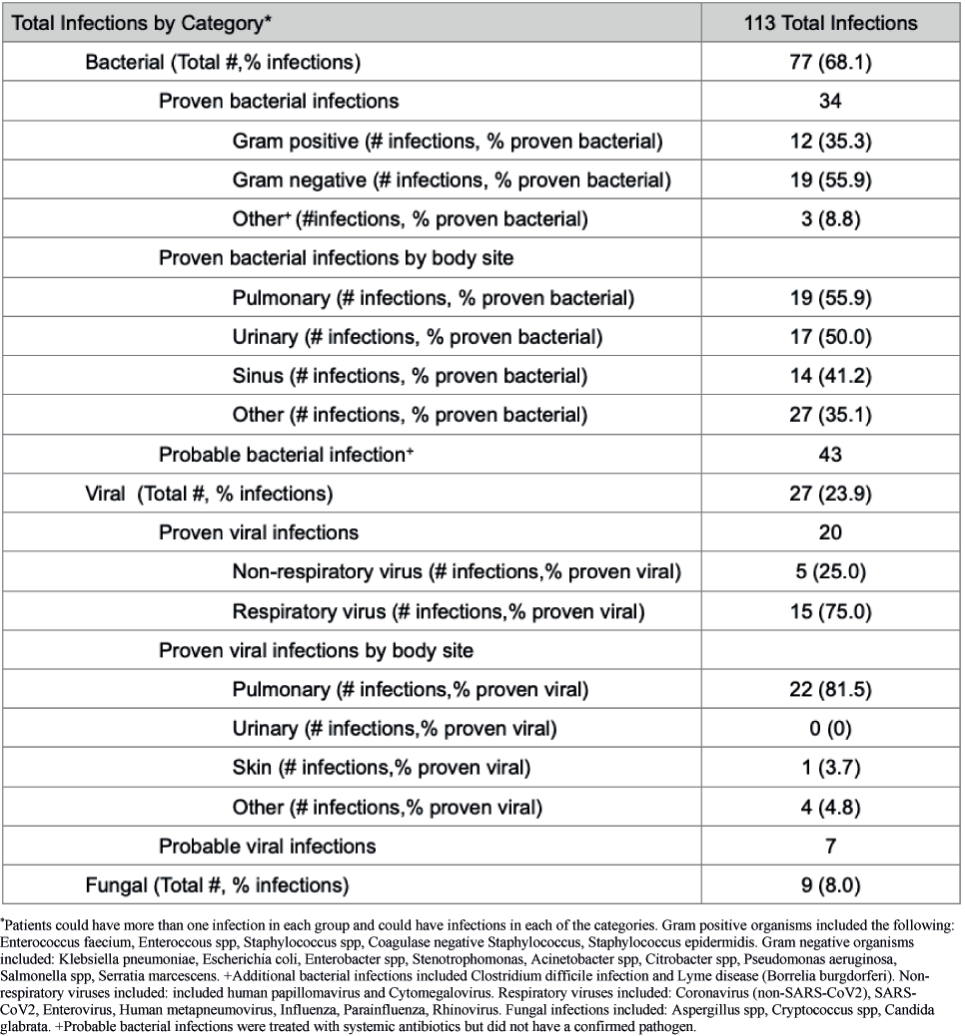

**Conclusion:**

Infectious complications, particularly of bacterial etiology, are common in the first year following CAR-T therapy. These data may inform future prophylactic strategies in this patient population.

**Disclosures:**

**Matthew Frigault, MD**, **Arcellx** (Consultant)**BMS** (Consultant)**Iovance** (Consultant)**Kite** (Consultant)**Novartis** (Consultant) **Jay A. Fishman, MD**, Nothing to disclose **Jon Arnason, MD**, **BMS/Juno** (Advisor or Review Panel member)**Regeneron** (Advisor or Review Panel member)

